# Toward a more comprehensive understanding of organizational influences on implementation: the organization theory for implementation science framework

**DOI:** 10.3389/frhs.2023.1142598

**Published:** 2023-08-31

**Authors:** Sarah A. Birken, Cheyenne R. Wagi, Alexandra G. Peluso, Michelle C. Kegler, Jure Baloh, Prajakta Adsul, Maria E. Fernandez, Manal Masud, Terry T-K Huang, Matthew Lee, Mary Wangen, Per Nilsen, Miriam Bender, Mimi Choy-Brown, Grace Ryan, Aliza Randazzo, Linda K. Ko

**Affiliations:** ^1^ Department of Implementation Science, Wake Forest University School of Medicine, Winston-Salem, NC, United States; ^2^ Emory Prevention Research Center, Rollins School of Public Health, Emory University, Atlanta, GA, United States; ^3^ College of Public Health, Department of Health Policy and Management, University of Arkansas for Medical Sciences, Little Rock, AR, United States; ^4^ Department of Internal Medicine, University of New Mexico, Albuquerque, NM, United States; ^5^ Comprehensive Cancer Center, University of New Mexico, Albuquerque, NM, United States; ^6^ Center for Health Promotion and Prevention Research, University of Texas Health Science Center at Houston School of Public Health, Houston, TX, United States; ^7^ Department of Health Systems and Population Health, School of Public Health, University of Washington, Seattle, WA, United States; ^8^ Center for Systems and Community Design and NYU-CUNY Prevention Research Center, Graduate School of Public Health and Health Policy, City University of New York, New York, NY, United States; ^9^ Department of Population Health, New York University Grossman School of Medicine, New York, NY, United States; ^10^ UNC Center for Health Promotion and Disease Prevention, University of North Carolina Chapel Hill, Chapel Hill, NC, United States; ^11^ Department of Health, Medicine and Caring Sciences, Division of Society and Health, Linköping University, Linköping, Sweden; ^12^ Sue & Bill Gross School of Nursing, University of California Irvine, Irvine, CA, United States; ^13^ College of Education and Human Development, School of Social Work, University of Minnesota, St. Paul, MN, United States; ^14^ Department of Population and Quantitative Health Sciences, University of Massachusetts Chan Medical School, Worcester, MA, United States

**Keywords:** organization theory, implementation, determinant framework, concept mapping, consensus-building

## Abstract

**Introduction:**

Implementation is influenced by factors beyond individual clinical settings. Nevertheless, implementation research often focuses on factors related to individual providers and practices, potentially due to limitations of available frameworks. Extant frameworks do not adequately capture the myriad organizational influences on implementation. Organization theories capture diverse organizational influences but remain underused in implementation science. To advance their use among implementation scientists, we distilled 70 constructs from nine organization theories identified in our previous work into theoretical domains in the Organization Theory for Implementation Science (OTIS) framework.

**Methods:**

The process of distilling organization theory constructs into domains involved concept mapping and iterative consensus-building. First, we recruited organization and implementation scientists to participate in an online concept mapping exercise in which they sorted organization theory constructs into domains representing similar theoretical concepts. Multidimensional scaling and hierarchical cluster analyses were used to produce visual representations (clusters) of the relationships among constructs in concept maps. Second, to interpret concept maps, we engaged members of the Cancer Prevention and Control Research Network (CPCRN) OTIS workgroup in consensus-building discussions.

**Results:**

Twenty-four experts participated in concept mapping. Based on resulting construct groupings' coherence, OTIS workgroup members selected the 10-cluster solution (from options of 7–13 clusters) and then reorganized clusters in consensus-building discussions to increase coherence. This process yielded six final OTIS domains: organizational characteristics (e.g., size; age); governance and operations (e.g., organizational and social subsystems); tasks and processes (e.g., technology cycles; excess capacity); knowledge and learning (e.g., tacit knowledge; sense making); characteristics of a population of organizations (e.g., isomorphism; selection pressure); and interorganizational relationships (e.g., dominance; interdependence).

**Discussion:**

Organizational influences on implementation are poorly understood, in part due to the limitations of extant frameworks. To improve understanding of organizational influences on implementation, we distilled 70 constructs from nine organization theories into six domains. Applications of the OTIS framework will enhance understanding of organizational influences on implementation, promote theory-driven strategies for organizational change, improve understanding of mechanisms underlying relationships between OTIS constructs and implementation, and allow for framework refinement. Next steps include testing the OTIS framework in implementation research and adapting it for use among policymakers and practitioners.

## Introduction

Individual healthcare providers' behaviors are often constrained by factors that are beyond their own control (1). The assumption that all behaviors are largely under conscious control has taken a “theoretical battering” due to research showing the importance of non-conscious processes that operate in organizations (2). Research suggests that many healthcare provider behaviors that are repeatedly performed become non-reflective and more or less automatic (3). Individual behavior is also constrained by factors at collective levels (1). Collective levels include interpersonal (e.g., relations between healthcare providers), group (e.g., healthcare professionals providing care in a breast medical oncology practice), intraorganizational (e.g., hospital culture), and interorganizational (e.g., accreditation standards). Collective-level influences may also be largely non-conscious, having become internalized and taken for granted (e.g., norms and values of a professional culture) (1).

Various implementation determinant frameworks include factors at the organizational level. For example, the Consolidated Framework for Implementation Research (CFIR) and the Exploration, Preparation, Implementation, Sustainment (EPIS) Framework include inner setting (i.e., intraorganizational) and outer setting (i.e., interorganizational) domain (4, 5). Domains are comprised of constructs (i.e., explanatory concepts that cannot be directly observed but can be inferred from observed data) (6). Organization-level domains include constructs such as *structural characteristics* (“the social architecture, age, maturity, and size of an organization”), *cosmopolitanism* (“the degree to which an organization is networked with other external organizations”), and funding (“fiscal support provided by the system in which implementation occurs”) (7–9). The Theoretical Domains Framework (TDF) similarly includes the environmental context and resources domain, which includes constructs such as *material resources* and *barriers and facilitators* (10).

Commonly used implementation determinant frameworks encourage implementation scientists to consider organizational influences on implementation; however, the scope of organization-level constructs described in extant frameworks is limited. Furthermore, determinant frameworks often lack explanations of the mechanisms underlying the relationships between organization-level constructs and implementation. Extant frameworks' limited scope impedes progress in implementation science by obscuring the influence of organization-level constructs that may drive implementation outcomes. A substantial body of work in industries other than healthcare provides evidence of the significant influence of organizational influences on implementation, pointing to high-leverage strategies to promote organizational change. Organization theory has been applied to educational and budgetary reform, elucidating the critical importance of addressing power dynamics among leadership and fostering positive change culture to facilitate implementation (11, 12). In the non-profit industry, organization theory can be used to build capacity, assist with decision-making, narrow target populations, and clarify organizational needs (13).

Failing to account for the critical influence of organization-level constructs on implementation introduces omitted variable bias–i.e., the faulty attribution of the influence of the omitted variable(s) to variables that were included (14). In the case of implementation research, this may amount to, for example, attributing the influence of organizational inertia (i.e., resistance to change) to a construct that is related but distinct (e.g., readiness for implementation) or an unrelated construct (e.g., individual provider motivation). The misattribution of omitted organization-level constructs to the constructs that extant implementation frameworks include has important implications for subsequent stages of implementation research, such as selecting and identifying strategies to target the constructs influencing implementation.

Many extant implementation determinant frameworks are conceptual frameworks, in that they offer a menu of constructs thought to influence implementation, but they do not address how change takes place or any causal mechanisms, which is critical for falsifying hypothesized relationships through empirical study (15). The ability to falsify hypothesized relationships between constructs depends on explanations of the mechanisms underlying relationships between constructs that are derived from theory (16). The constructs in conceptual frameworks such as the CFIR derive from a combination of theory and empirical studies. For example, the CFIR *peer pressure* construct derives from Institutional Theory, but *patient needs and resources* derives from a combination of empirical evidence and other conceptual frameworks rather than theory.

In contrast to conceptual frameworks, theoretical frameworks are based on theories, which propose mechanisms underlying the relationship between constructs and implementation. One commonly used theoretical framework in implementation science is the TDF. As a theoretical framework, the TDF can be used to identify mechanisms proposed in included theories; however, the TDF does not offer nuanced insight into organization-level influences on implementation. The TDF's environmental context and resources domains contains constructs that derive from several theories that are identified as organization theories; however, many of the included theories are not in fact organization theories (e.g., decision-making theory). As such, the TDF is limited in its contributions to understanding organization-level influences on implementation.

Organization theories provide explanations for the complex interactions within and between organizations and their context (environment, surrounding policies, cultural norms). These theories not only describe and explain these interactions, but can also be used to predict implementation outcomes based on contextual factors. Organization theories have the potential to explain how policies, institutions, funding, and workforce dynamics affect implementation outcomes (17). Organization theories have been historically used to an explanatory tool in fields of education, nonprofit organizations, management, and health services research, dating back to the 1950 s (11–13, 18, 19). These theories, while widely used and published, remain largely inaccessible outside of organization science. Organization theories provide their own inventory of constructs, which often require significant training to apply with fidelity.

To equip implementation scientists with understanding of a broader scope of organization-level constructs and their hypothesized influence on implementation, a comprehensive yet accessible framework of organizational influences on implementation is needed. In this paper, we describe the development of the Organization Theory for Implementation Science (OTIS) framework, which summarizes constructs from nine organization theories identified as relevant to implementation in preliminary studies (20). Our overarching goal is to increase implementation scientists' familiarity with and conceptualization of the myriad organizational factors that influence implementation through mechanisms clearly articulated by organization theories.

## Materials and methods

We developed the OTIS framework using a combination of concept mapping and iterative consensus-building, with support for interpretation from members of the Cancer Prevention and Control Research Network (CPCRN) OTIS workgroup (21). CPCRN is a national network of academic, public health, and community partners whose work focuses on reducing the burden of cancer within specific workgroup and interest group projects. CPCRN OTIS workgroup members include investigators conducting research at the intersection of implementation science and cancer prevention and control. This study was approved by the Wake Forest University School of Medicine IRB (IRB00072134) on 6/2/21.

### Concept mapping

#### Recruitment and sampling

We used a purposive sampling approach to recruit approximately 25 scholars with expertise at the intersection of implementation and organization science to participate in an online concept mapping exercise via the Concept Systems Global MAX™ web platform (22). The premise of our sampling approach for the survey on organization theories of relevance to implementation science that provided the foundation for this study was that scholars with primary training in implementation and organization science had the knowledge required to identify organization theories with relevance to implementation science. For this study, we purposively included a more diverse group of scholars with implementation and organization expertise with the objective of generating a framework that would reflect the perspective of targeted users of the OTIS framework. Between 20 and 30 sorters have been found to maximize concept mapping fit consistency, yielding results similar to concept mapping by several hundred participants (23). Members of the study team identified potential participants from their respective professional networks in Canada, the UK, and the USA, as well as professional organizations such as the VA QUERI Implementation Research Group. We sent up to three emails offering potential participants a $50 incentive to engage in the concept mapping exercise.

#### Procedure

To identify conceptually distinct categories (domains) of constructs, we asked participants to sort virtual cards for each of the 70 constructs from nine organization theories relevant to implementation identified in previous work (20), accompanied by their definitions, into piles as they deemed appropriate. We then asked participants to name each pile. Participants could engage in the activities in the order of their choosing and could do so over multiple online sessions, at their convenience, until their responses were complete.

#### Analysis

Data analysis involved the use of multidimensional scaling and hierarchical cluster analyses to produce visual representations of the relationships among the constructs (23). Specifically, multidimensional scaling was used to generate a point map depicting each of the constructs and the relationships between them based upon a summed square similarity matrix. Constructs frequently sorted together were placed closer together on the point map (23). Hierarchical cluster analysis was used to partition the point map into non-overlapping clusters (i.e., domains) (23). The Concept Systems Global Max™ suggested potential cluster labels based upon participant responses. Model fit was assessed using the stress value, an indicator of goodness of fit between the point map and the total similarity matrix. Cross-study syntheses of concept mapping studies have consistently found mean stress values of 0.28 (24). The stress value of the concept map represents goodness of fit of the configuration, demonstrating how close the solution is to the original groupings made by the participants. Lower stress values indicate a better fit than higher stress values (24).

### Consensus-building

#### Recruitment and sampling

We invited members of the CPCRN OTIS workgroup to review concept mapping results and provide feedback. All CPCRN OTIS workgroup members were eligible to participate.

#### Procedure

To build upon the results of the concept mapping activity, CPCRN OTIS workgroup members provided their expertise in reviewing results of the concept mapping activity. Participation occurred over the course of three months, beginning with the CPCRN Annual Meeting and continuing through regular workgroup meetings.

#### Analysis

During a hybrid meeting held in May 2022, CPCRN OTIS workgroup members (6 in-person; 4 virtual) considered a range of potential cluster solutions, ranging from seven to 10 clusters, to determine which solution best suited the purposes of the current study. Each member identified the cluster map that they deemed most conceptually clear based on their knowledge of the field. The group then discussed their choices and worked to reach consensus on what the group believed to provide the most conceptually clear map and moved constructs to clusters that provided the best fit. The group also discussed and altered the automatically generated labels created by Global Max™. Following the initial analysis, two workgroup members reviewed notes, and a third member reconciled discrepancies, suggesting additional shifts of constructs among clusters. Finally, the lead investigator revised clusters based on extensive knowledge of organization theory. The resulting clusters were again reviewed, revised, and approved by CPCRN OTIS workgroup members during workgroup meetings until a consensus was reached.

## Results

### Concept mapping

Twenty-four scholars participated in the concept mapping exercise. Participant demographics are described in Table 1. Most participants (84%) held a PhD degree and worked in an academic institution (80%). The plurality of participants had multidisciplinary training (29%), and the majority had multidisciplinary expertise (57%).

**Table 1:** Concept mapping participant demographics.

Characteristic	Percent Total
	*N* = 25
Education	*N* (%)
PhD	21 (84.0)
MD	1 (4.0)
Other	3 (12.0)
Academic Title	*N* = 24
Assistant professor	6 (25.0)
Post-doctoral fellow	5 (20.8)
Professor	5 (20.8)
Associate professor	4 (16.7)
Other	2 (8.3)
Did not respond	2 (8.3)
Organization	*N* = 24
University	19 (80.0)
Government Agency	2 (8.0)
Other	2 (8.0)
Research Institute	1 (4.0)
Field, Specialty, or Discipline	*N* = 24
Multidisciplinary	7 (29.2)
Health policy and management	3 (12.5)
Implementation science	3 (12.5)
Social work	2 (8.3)
Behavioral science/public health	2 (8.3)
Health care management	1 (4.2)
Health services research	1 (4.2)
Sociology	1 (4.2)
Clinical psychologist	1 (4.2)
Behavioral science	1 (4.2)
Healthcare	1 (4.2)
Organizational behavior	1 (4.2)
Content Expertise	*N* = 23
Multidisciplinary	13 (56.5)
Cancer (prevention, control, survivorship)	4 (17.4)
Mental health	2 (8.7)
Health services research	1 (4.3)
Health and social care	1 (4.3)
Digital technology in healthcare	1 (4.3)
Maternal and child health	1 (4.3)

All 24 participants completed the sorting exercise. We confirmed that sorts were valid by checking 5 participants' responses to ensure that criteria were sorted into generally logical categories. The stress value was 0.32, demonstrating poor fit. Our consensus-building process was designed to address poor fit by developing a more coherent solution.

### Consensus-building

**Figure 1:**
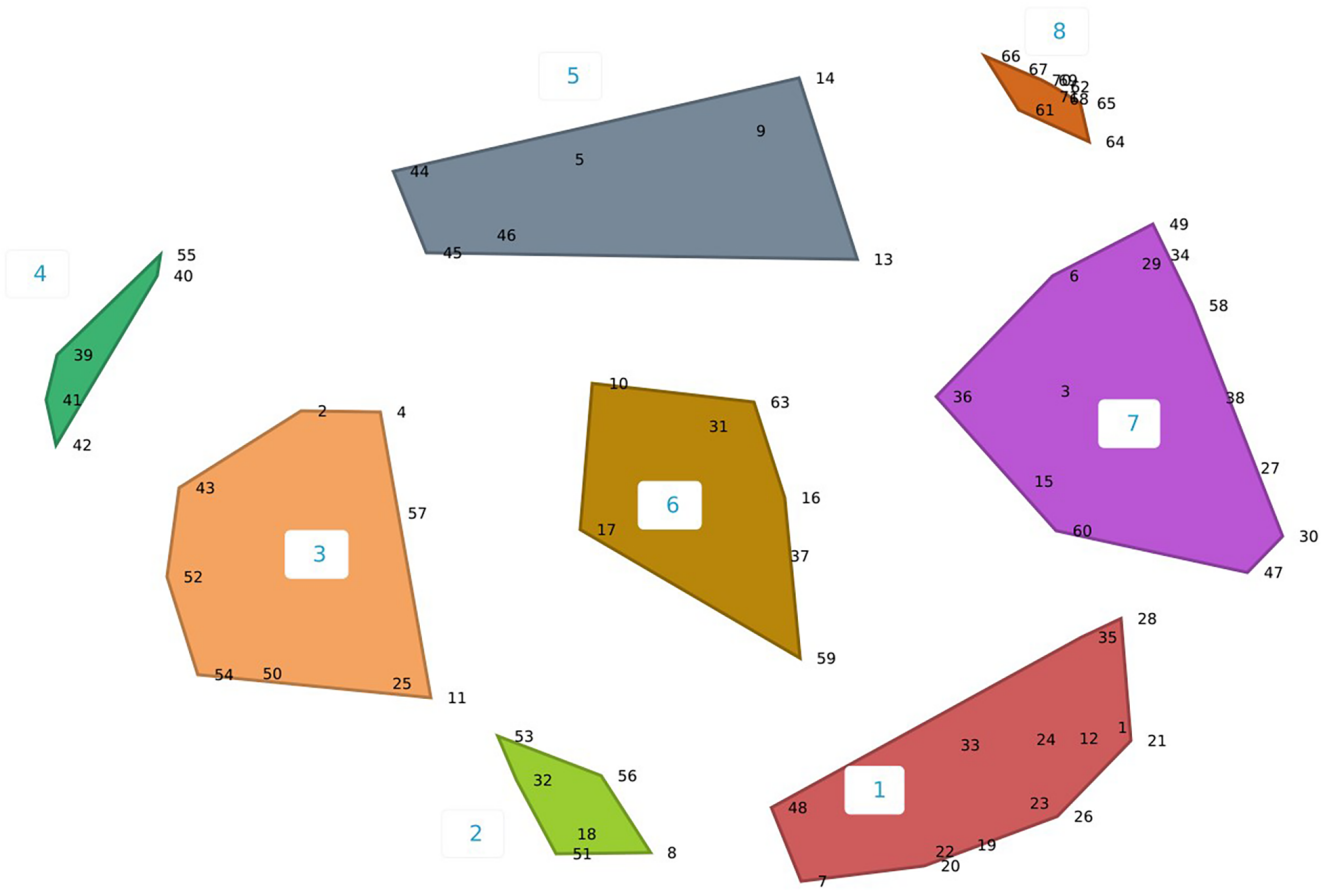
Eight-cluster concept map solution.

**Figure 2:**
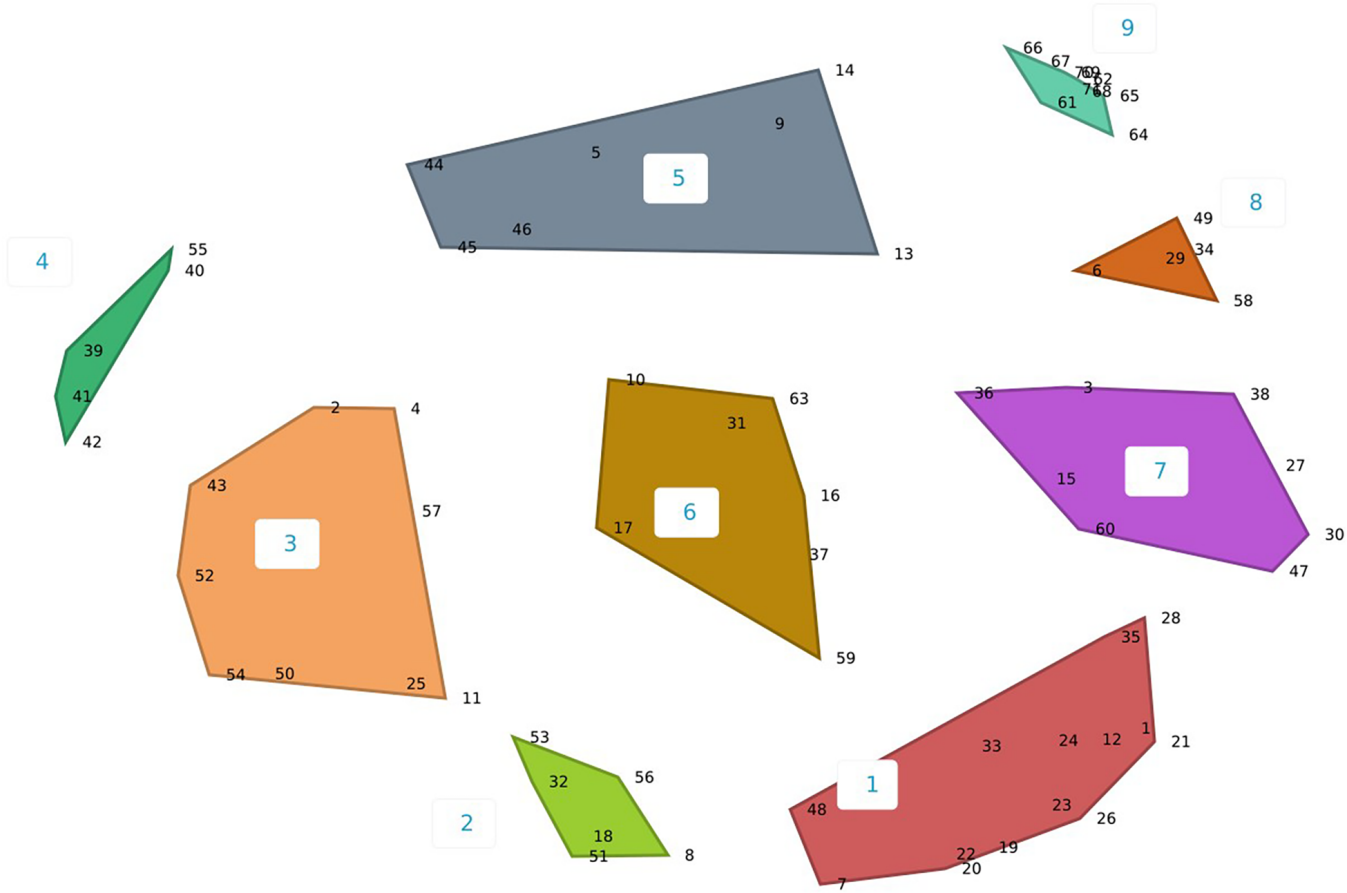
Nine-cluster concept map solution.

**Figure 3:**
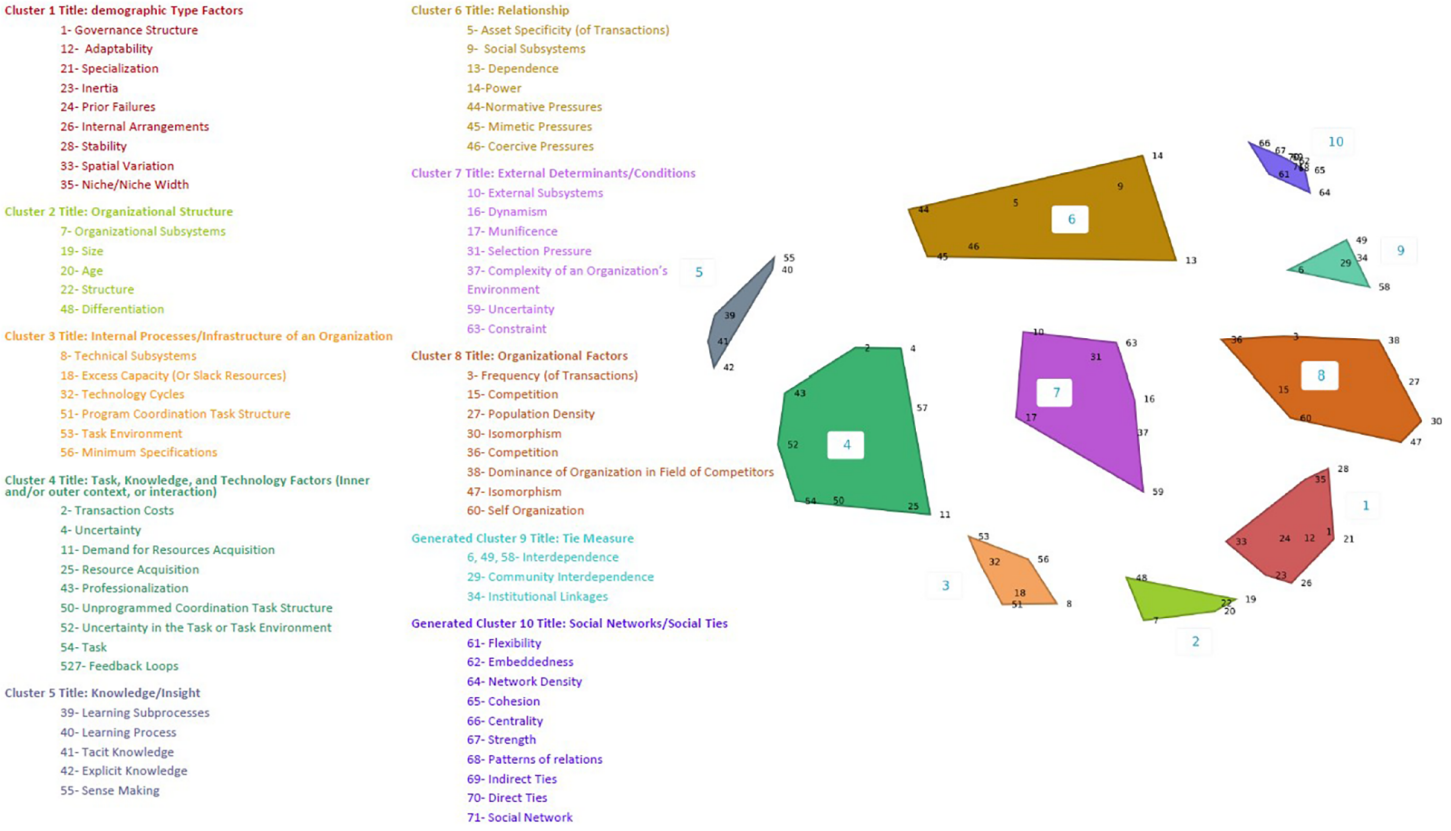
Ten-cluster concept map solution and constructs.

Workgroup members (*n* = 18) participated throughout in-person and virtual discussion sessions. Participant demographics are described in Table 2. The concept mapping software produced multiple cluster options, ranging from 7 to 13 clusters. CPCRN OTIS workgroup members narrowed the clusters to 8–10 (Figures 1–3), ultimately selecting the 10-cluster solution to use as a starting point for the consensus-building process. Workgroup members then reorganized the clusters to increase coherence, yielding six final OTIS framework domains: organizational characteristics; governance and operations; characteristics of a population of organizations; tasks and processes; knowledge and learning; and interorganizational relationships. The final solution was informed by the 10-cluster solution. A total of 70 constructs are organized across the six domains. Supplementary File S1 organizes constructs by domain and includes brief descriptions/definitions for each, as well as the source theory.

**Table 2:** Consensus gathering participant demographics.

Characteristic	Total
	*N* = 18
Education	*N* (%)
PhD	13 (72.2%)
MD	0
Other	5 (27.8%)
Academic Title	*N* = 18
Assistant professor	4 (22.2%)
Post-doctoral fellow	1 (5.6%)
Professor	5 (27.8%)
Associate professor	3 (16.7%)
Other	5 (27.8%)
Organization	*N* = 18
University	18 (100%)
Government Agency	0
Other	0
Research Institute	0
Field, Specialty, or Discipline	*N* = 18
Multidisciplinary	5 (27.8%)
Health policy and management	0
Implementation science	7 (38.9%)
Social work	1 (5.6%)
Behavioral science/public health	5 (27.8%)
Health care management	0
Health services research	0
Sociology	0
Clinical psychologist	0
Behavioral science	0
Healthcare	0
Organizational behavior	0
Content Expertise	*N* = 18
Multidisciplinary	6 (33.3%)
Cancer (prevention, control, survivorship)	8 (44.4%)
Mental health	1 (5.6%)
Health services research	3 (16.7%)
Health and social care	0
Digital technology in healthcare	0
Maternal and child health	0

*Organizational Characteristics* (number of constructs = 6) refers to the features of an organization that may predispose it to governance, operations, interorganizational relationships, etc. Included constructs relate to change dynamics (e.g., inertia; adaptability), orientation to operations (e.g., professionalization; specialization); and dominance within its population [e.g., age; size (i.e., indicators of viability; on average, older, larger organizations are more likely to survive than younger, smaller organizations)].

*Governance and Operations* (*n* = 7) refer to the rules and operating procedures that govern an organization. An organization's rules and operating procedures may be established explicitly (e.g., intentionally, by a governing body) or implicitly (e.g., passively, through repeated operations). Constructs include approaches to operating (e.g., governance structure; internal arrangements) and structures that characterize an organization's operations (e.g., internal arrangements; feedback loops).

*Characteristics of a Population of Organizations* (*n* = 16) refer to the features of a group of organizations of which the referent organization is a member (25). The institutions that comprise an organization's population may vary depending on the objective or problem in question. That is, a referent organization may be part of several populations. For example, a hospital's population may be defined as local healthcare organizations with respect to competition for physicians and patients, but with respect to adherence to government regulations, a hospital's population may be defined as all of the country's hospitals. Constructs included in the *Characteristics of a Population of Organizations* domain are features of the population as a whole rather than features of the organizations that comprise the population. Constructs relate to change within the population (e.g., dynamism; stability); competition (e.g., competition; selection pressure); variation within the population (e.g., isomorphism; spatial variation); and availability of resources (e.g., munificence; constraint).

*Tasks and Processes* (*n* = 16) characterize the work that an organization pursues and the conditions that influence its approach to accomplishing the work. Included constructs refer to features of the processes used to accomplish tasks (e.g., un/programmed coordination task structure; transaction costs); features of the environment in which tasks are accomplished (e.g., dependence; excess capacity); and features of the task (e.g., frequency of transactions; technology cycles).

*Knowledge and Learning* (*n* = 5) refers to the information available to an organization in pursuing its goals and the processes used to acquire the information. Included constructs relate to characteristics of knowledge (e.g., tacit and implicit knowledge) and approaches to acquiring knowledge (e.g., learning (sub)processes; sense making).

*Interorganizational Relationships* (*n* = 20) refer to characteristics of the interactions that an organization has with other institutions. In contrast to the *Characteristics of a Population of Organizations* domain, which refers to features of a population of organizations as a whole, the *Interorganizational Relationships* domain characterizes communal (e.g., communication) or exchange (e.g., monetary or other resource exchange) interactions (26). Included constructs characterize an organization's dependence on other institutions (e.g., interdependence; community interdependence); the pressure that organizations exert on each other (e.g., normative, mimetic, and coercive pressure; dominance; power).

## Discussion

This study describes how we created the OTIS framework to increase implementation scientists' familiarity with and conceptualization of the diverse set of organizational influences on implementation. Increasing implementation scientists' conceptualization of organizational influences may contribute to more comprehensive understanding of the key drivers of implementation and, in turn, our ability to identify and select strategies to accelerate the translation of evidence into practice, as found in other industries, such as business and education (11, 12, 17, 18). Our efforts yielded six conceptually distinct domains, encompassing 70 constructs from nine organization theories with relevance to implementation. Distilling many constructs from several theories into a limited number of domains limits the burden on implementation scientists to account for the vast array of potentially important organizational influences on implementation. The six domains that we identified in this study reflect concepts that are central to organization theory, including power, structure, autonomy, control (20), but which are less commonly addressed in implementation science. The concepts reflected in the OTIS framework offer perspective on key questions in implementation science, such as how and why organizations adopt, implement, and sustain evidence-based practices—or resist doing so.

The OTIS framework considerably expands upon existing implementation determinant frameworks' conceptualization of organizational influences on implementation. OTIS includes constructs such as *specialization*, which is not explicitly captured in the CFIR or EPIS frameworks, but which may influence the decision to adopt an evidence-based practice. For example, a study of determinants of low-value use of computed tomography to evaluate microscopic hematuria found that, while urologists' evaluation practices changed following the American Urological Association's revised guidelines, primary care providers' evaluation practices often went unchanged, highlighting the need to tailor strategies for the various specialties involved in implementation (27, 28). OTIS greatly expands upon the TDF's organization-level *environmental context and resources* domain with more nuanced domains, such as *interorganizational relationships*. Future efforts should systematically map OTIS onto extant determinant frameworks to clearly articulate OTIS's unique contribution. For example, OTIS's Governance and Operations and Tasks and Processes domains include several constructs that may add critical nuance to EPIS's Funding/Contracting construct. Before systematic mapping of OTIS onto extant frameworks, OTIS may be used in its current form in conjunction with other frameworks, such as the CFIR and TDF, which are increasingly used in combination and already capture intra-organizational constructs, such as climate and leadership (28). For example, some OTIS domains or constructs that appear not to be captured in CFIR (e.g., tasks and processes; stability of the population of organizations; normative pressures) could be included in implementation determinant studies.

OTIS also expands upon commonly used implementation frameworks by allowing users to access organization theories articulate the mechanisms underlying relationships between included constructs and implementation. For example, the EPIS framework identifies sociopolitical influences on implementation (e.g., legislation; monitoring and review); however, EPIS does not articulate *how* or *why* these constructs influence implementation. In contrast, OTIS's basis in theory allows users to identify hypothesized relationships between included constructs and implementation, as clearly articulated in publicly available OTIS abstraction forms (28). Specifically, users may consult the *propositions* section of OTIS abstraction forms to identify mechanisms underlying included constructs. For example, OTIS describes how coercive influences of governments and accrediting bodies exert normative pressure (Interorganizational Relationships domain) on healthcare organizations to comply with legislation and monitoring by virtue of organizations' dependence on these governing bodies for permission to operate. Therefore, OTIS could be used in conjunction with extant frameworks to explain the mechanisms underlying constructs' influence on implementation (29). Clearly articulated mechanisms are critical for identifying strategies that are best-suited to influence the construct identified as influencing implementation.

Members of the CPCRN OTIS workgroup are currently applying the OTIS framework in the following projects: Project 1 a) tests the conceptual validity and applicability of the OTIS framework in community oncology practices and b) develops, tests, and disseminates tools using OTIS in implementation research, including a qualitative interview guide and codebook. Project 2 is an American Society of Clinical Oncology collaborative study on Sexual Orientation and Gender Identity data collection. OTIS will be used in this project to a) reanalyze data that have been previously analyzed using the CFIR, and b) compare results between CFIR and OTIS findings. Project 3 applies OTIS to a CDC-funded U01 cooperative agreement to reduce health inequities for cancer survivors in the District of Columbia. OTIS will be used to a) to build community coalitions of approximately 10 organizations to improve infrastructure and communication and b) to think consider power dynamics and the elimination of disparities and health inequalities. Some limitations of our study should be noted. Concept mapping requires participants to have pre-existing knowledge and experience with the topic they are mapping, limiting the pool of potential participants. There is a limited population of researchers with the required familiarity of organization theories and implementation science to participate in concept mapping. As a result, our purposive sampling approach was necessary to increase the likelihood that participants would understand included constructs enough to sort and rate them. However, it is possible that participants lacking refined expertise in implementation or organization science would have valuable perspective on included constructs. For example, hospital administrators may lack fluency in the terminology included in organization theories, but they may have unique insight into how, for example, normative pressure from professional organizations influences implementation. Future work should refine the language used in OTIS to increase its accessibility to an audience without expertise in organization science. Additionally, the clusters that Global Max generated, in many cases, lacked coherence as indicated by the stress value of 0.32, suggesting variation in concept mapping participants' interpretation of the constructs and their relationships. To address this concern, OTIS workgroup members used their expertise to reorganize many clusters in our consensus-building process, potentially suggesting the limited utility of concept mapping for developing the framework. We view the OTIS framework as a living document to be revised through application. For example, implementation scientists may find through qualitative interview data collection that study participants describe OTIS constructs in combinations not reflected in the domains identified in this study. Future iterations of the OTIS framework will be revised to reflect empirical evidence.

Despite these limitations and the need for continued development, OTIS may be used in its current form in implementation research. OTIS could be used to inform data collection or analysis. For example, OTIS could be used to develop guides for interviews with cancer program leadership to understand the potential influence of participation in quality improvement networks, professional norms, and the ability to recruit providers influence compliance with cancer program accreditation standards (30). We plan to use OTIS to analyze data that were previously collected regarding factors influencing cancer programs' implementation of exercise interventions. In each of these cases, OTIS offers researchers the tools necessary to understand the mechanisms underlying factors that influence implementation, pointing toward strategies to facilitate implementation (e.g., strengthening or reorganizing quality improvement networks to support compliance with accreditation standards).

## Conclusions

We distilled 70 constructs from nine organization theories into six domains in the OTIS framework. The OTIS framework has several potential benefits. First, OTIS may enhance implementation scientists' consideration of organization-level constructs, which to date has been insufficient (17). Second, OTIS adds nuance to relatively limited conceptualizations of organizational influences in extant implementation determinant frameworks, such as the CFIR, EPIS, and TDF. Third, OTIS may increase the use of theories in implementation science. Evidence suggests that the use of theories, models, and frameworks in implementation science is inconsistent and often inappropriate (31). Unlike conceptual frameworks, which offer a menu of constructs thought to influence implementation, theoretical frameworks including OTIS are based on theories, which propose mechanisms underlying the relationship between constructs and implementation. OTIS links implementation scientists to theories that may explain the phenomena underlying complex implementation problems, such as slow uptake or poor sustainment. Future efforts should include expanding extant frameworks with OTIS's unique domains and constructs; refining OTIS's language to increase its accessibility to an audience without expertise in organization science; and revising OTIS to reflect empirical evidence.

## Data Availability

The raw data supporting the conclusions of this article will be made available by the authors, without undue reservation by reasonable request to the corresponding author.
