# Supplementary material for: Toward a more comprehensive understanding of organizational influences on implementation: the organization theory for implementation science framework
Source: Front Health Serv. 2023 Aug 31;3:1142598. doi: 10.3389/frhs.2023.1142598 (PMC10501605; doi:10.3389/frhs.2023.1142598)
Supplement: Supplementary file 1 [file Datasheet1.docx]

| Additional File 1. OTIS Domains and Constructs | |  |
| --- | --- | --- |
| Domain | Constructs | Source Theory |
| Organizational Characteristics - refers to features of an organization that may predispose it to approaches to governance, operations, interorganizational relationships, etc. Included constructs relate to change dynamics (e.g., inertia), orientation to operations (e.g., professionalization); and dominance within its population (e.g., age; size [i.e., indicators of viability; on average, older, larger organizations are more likely to survive than younger, smaller organizations). | AGE - The length of an organization’s life history | Population Ecology |
|  | INERTIA - Organizational resistance to change | Population Ecology |
|  | PRIOR FAILURES - Previous deterioration(s) in an organization’s adaptation to its small niche and the associated reduction of resources within the organization | Population Ecology |
|  | PROFESSIONALIZATION - Claims on knowledge among professional groups | Population Ecology |
|  | SIZE - The capacity to carry interactions among resources, constraints and demand | Population Ecology |
|  | STRUCTURE - An organization’s goals, authority, strategy, core technology | Institutional Theory |
| **Governance and Operations -** Governance and operations refers to the rules and operating procedures that govern an organization. An organization’s rules and operating procedures may be established explicitly (e.g., intentionally, by a governing body) or implicitly (e.g., passively, through repeated operations). Constructs include approaches to operating (e.g., governance structure; internal arrangements) and structures that characterize an organization’s operations (e.g., internal arrangements; feedback loops). | ADAPTABILITY - Ability of an organization to change in an attempt to address environmental demands | Resource Dependency Theory |
|  | FEEDBACK LOOPS - A phenomenon characterized by outputs of a system continuously becoming the inputs | Complexity Theory |
|  | GOVERNANCE STRUCTURE - A continuum of approaches to generating a desired product or service ranging from buying it from another party to making it yourself: Spot market is when organization buys with no contract (i.e., open market); "Hybrid" contracting modes are when organization buys with a contract, and may include long-term commercial contracts, informal agreements, and franchise contracting, exclusive dealing contract; Fully integrated firm is when the organization makes the product itself, by unifying ownership and control | Transaction Cost Economics |
|  | INTERNAL ARRANGEMENTS - Actions and factors within an organization (e.g., internal politics) | Population Ecology |
|  | ORGANIZATIONAL SUBSYSTEMS - Infrastructure, leadership and management, resources, teamwork and communication, organizational readiness for change, organizational context | Sociotechnical Theory |
|  | SOCIAL SUBSYSTEMS - Attributes of people (i.e., skills, attitudes, concerns, expectation, and values); relationships among people; reward systems; and authority structure | Sociotechnical Theory |
|  | SPECIALIZATION - The restricted niche breadth/area of a given organization | Population Ecology |

| **Characteristics of A Population of Organizations -** Population characteristics refers to the collective features of the group of institutions in the referent organization’s environment. The institutions that comprise an organization’s population may vary depending on the objective or problem in question. For example, a hospital’s population may be defined as local healthcare organizations with respect to competition for physicians and patients, but with respect to adherence to government regulations, a hospital’s population may be defined as all of the country’s hospitals. Included constructs characterize the collective features of the population rather than the features of the organizations that comprise the population. Constructs relate to change within the population (e.g., dynamism; stability); competition (e.g., competition; selection pressure); variation within the population (e.g., isomorphism; spatial variation); and availability of resources (e.g., munificence; constraint). | COMPETITION - The number and diversity of stakeholders (competitors, suppliers, and buyers) that an organization needs to consider in formulating strategies; perceptions that another organization in the field poses a threat | Resource Dependency Theory |
| --- | --- | --- |
|  | COMPETITION - A process by which “(1) demand for resources exceeds supply; (2) competitors become more similar as standard conditions of competition produce a uniform response; (3) selection eliminates the weakest competitors; and (4) deposed competitors differentiate either territorially or functionally, yielding a more complex division of labor” | Population Ecology |
|  | COMPLEXITY OF AN ORGANIZATION'S ENVIRONMENT - The extent to which the context in which an organization operates is or is not (1) stable over time and (2) predictable (e.g., customer preferences; availability of resources) | Organization Learning |
|  | CONSTRAINT - A linkage or other restriction that becomes a limitation and/or an inhibition | Network Perspective |
|  | DYNAMISM - The rate of environmental change or innovation in the external environment | Resource Dependency Theory |
|  | EXTERNAL SUBSYSTEMS - Outside forces and influences on an organization (e.g., stakeholders; regulations) | Sociotechnical Theory |
|  | ISOMORPHISM - Similar organizational structures and processes | Institutional Theory |
|  | ISOMORPHISM - A similarity of processes or structure among organizations | Population Ecology |
|  | MUNIFICENCE - The availability and accessibility of resources necessary for an organization’s development and survival within the external environment | Resource Dependency Theory |
|  | NICHE/NICHE WIDTH - (The size of) An area in a constraint space in which a population can survive and reproduce itself | Population Ecology |
|  | POPULATION DENSITY - The number of organizations in a population (i.e., group of organizations that is distinguishable from other groups) | Population Ecology |
|  | SELECTION PRESSURE - External agents that affect an organization’s ability to survive in a given environment | Population Ecology |
|  | SPATIAL VARIATION - Different values of organizational characteristics across locations | Population Ecology |
|  | STABILITY - The extent to which conditions change over time | Population Ecology |
|  | UNCERTAINTY - The unpredictability of a system’s behavior and its effects | Complexity Theory |
|  | UNCERTAINTY - The extent to which changes to the wider environment may influence transactions and the future actions of transacting parties are unknown | Transaction Cost Economics |

| **Tasks and Processes -** Tasks and processes characterize the work that an organization pursues and the conditions that influence its approach to accomplishing the work. Included constructs refer to features of the processes used to accomplish tasks (e.g., un/programmed coordination task structure; transaction costs); features of the environment in which tasks are accomplished (e.g., dependence; excess capacity); and features of the task (e.g., frequency of transactions; technology cycles). | ASSET SPECIFICITY (OF TRANSACTIONS) - The degree to which transacting parties have invested transaction-specific human, physical, or other forms of capital specific to the transaction (e.g., additional training, equipment, and staff) | Transaction Cost Economics |
| --- | --- | --- |
|  | DEMAND FOR RESOURCES ACQUISITION - An organization’s need to acquire resources from the external environment to sustain its internal environment | Resource Dependency Theory |
|  | DEPENDENCE - The extent that an organization relies on another organization to obtain resources that it requires to exist (e.g., material, human resources, legitimacy); the obverse of power | Resource Dependency Theory |
|  | DIFFERENTIATION- The extent to which, within an organization, different parts/departments perform different tasks and have different relevant sub-environments. | Contingency Theory |
|  | EXCESS CAPACITY (OR SLACK RESOURCES) - Production at a lower scale of output than it has been designed for | Population Ecology |
|  | FREQUENCY (OF TRANSACTIONS) - How often a transaction occurs | Transaction Cost Economics |
|  | MINIMUM SPECIFICATIONS - A few, flexible, simple rules: direction pointing (accounting for past phenomena in future iterations); boundaries (delimitations of the system); resources (means available); permissions (latitude in decision-making) | Complexity Theory |
|  | PROGRAM COORDINATION TASK STRUCTURE - The activities involved in completing a task are specified and codified in advance via (1) rules and programs (i.e., standardization) and (2) centralization of decision making and authority arrangements | Contingency Theory |
|  | RESOURCE ACQUISITION - The process by which new organization(s) acquire resources | Population Ecology |
|  | TASK - The work that is performed | Contingency Theory |
|  | TASK ENVIRONMENT - The context where work is performed (both the organizational setting and its wider, socio-political-economic context) | Contingency Theory |
|  | TECHNICAL SUBSYSTEMS - Technologies, techniques, tasks performance, methods and work setting; features include data cleansing and migration, features and functionalities of application, adaptability and flexibility or new system, system benefits, usability, stability | Sociotechnical Theory |
|  | TECHNOLOGY CYCLES - A sequence of processes that involve technology (i.e., the means, activities, and knowledge to transform materials and inputs into outputs; e.g., human resources) | Population Ecology |
|  | TRANSACTION COSTS - The outlay required for contract negotiations, monitoring adherence to contractual terms, providing financial incentives or penalties, and losses resulting from supplier noncompliance | Transaction Cost Economics |
|  | UNCERTAINTY IN THE TASK OR TASK ENVIRONMENT - The gap between the amount of information that is needed and the amount of information that is available to achieve a given level of performance on a task | Contingency Theory |
|  | UNPROGRAMMED COORDINATION TASK STRUCTURE - The activities involved in completing a task are not specified in advance by the organization; activities are worked out by organization members via (1) professionalization deferring to expertise, (2) providing additional time and resources for collaboration, (3) creation of self-contained tasks, (4) providing real-time data to frontline individuals and teams, and (5) promoting and supporting horizontal coordination and communication | Contingency Theory |
| **Knowledge and Learning -** Knowledge and learning refer to the information that an organization has to wield in pursuing its goals and the processes used to acquire the information. Included constructs relate to characteristics of knowledge (e.g., tacit and implicit knowledge) and approaches to acquiring knowledge (e.g., learning (sub)processes; sense making). | EXPLICIT KNOWLEDGE - Facts and information that can be codified (e.g., in policies and procedures) | Organizational Learning |
|  | LEARNING PROCESS - An interaction of experience (history) and context that produces knowledge | Organizational Learning |
|  | LEARNING SUBPROCESSES - A series of actions associated with the learning process, including: Knowledge creation, Knowledge transfer, Knowledge retention, Knowledge search | Organizational Learning |
|  | SENSE MAKING - A social activity through which people assign meaning to experience | Complexity Theory |
|  | TACIT KNOWLEDGE – Facts, information, and skills that are difficult to codify | Organizational Learning |
| **Interorganizational Relationships** - Interorganizational relationships refer to characteristics of the interactions that an organization has with other institutions. Included constructs characterize an organization’s dependence on other institutions (e.g., interdependence; community interdependence); the pressure that organizations exert on each other (e.g., normative, mimetic, and coercive pressure; dominance; power); and characteristics of the linkages between an organization and other institutions (e.g., (in)direct ties; strength, cohesion, and centrality of ties). | CENTRALITY - The importance of an actor’s position in a network structure (e.g., prominence of opinion leaders) | Network Perspective |
|  | COERCIVE PRESSURES - “Formal and informal pressures exerted on organizations by other organizations upon which they are dependent and by cultural expectations in the society within which organizations function" | Institutional Theory |
|  | COHESION - The connectedness or “knittedness” of a network | Network Perspective |
|  | COMMUNITY INTERDEPENDENCE - The extent to which interactions among co-acting sets of organizational/community populations depend on each other | Population Ecology |
|  | DIRECT TIES - Connections in which a single tie spans two actors | Network Perspective |
|  | DOMINANCE OF ORGANIZATION IN FIELD OF COMPETITORS - The extent to which an organization is perceived to be powerful in relation to its competitors | Organizational Learning |
|  | EMBEDDEDNESS - The extent that social ties are forged, renewed, and extended through the community rather than through actors outside the community | Network Perspective |
|  | FLEXIBILITY - The extent that social ties are forged, renewed, and extended through the community rather than through actors outside the community | Network Perspective |
|  | INDIRECT TIES - Connections where ties exist between actors but only through other actors | Network Perspective |
|  | INSTITUTIONAL LINKAGES - Relationships created between organization(s) for a cause | Population Ecology |
|  | INTERDEPENDENCE - The relationships, connections, and interactions among the parts of a complex system | Complexity Theory |
|  | INTERDEPENDENCE- To what degree/extent different actors must interact to complete work. | Contingency Theory |
|  | INTERDEPENDENCE - The interaction among social subsystems, technical subsystems, and organizational subsystems | Sociotechnical Theory |
|  | MIMETIC PRESSURES - Influences encouraging organizations to model the behavior of other organizations in their field | Institutional Theory |
|  | NETWORK DENSITY - A measure of cohesion expressed as the number of ties in a network divided by the maximum number of ties that are possible | Network Perspective |
|  | NORMATIVE PRESSURES - Influences derived from members of an occupation or profession (e.g., physicians) defining the conditions and methods of work | Institutional Theory |
|  | PATTERNS OF RELATIONS - Patterns of ties that yield a particular network structure (e.g., structural holes) | Network Perspective |
|  | POWER - Dominance in a relationship; the obverse of dependence | Resource Dependency Theory |
|  | SOCIAL NETWORK - A set of actors (e.g., individuals, organizations) connected by one or more social ties (e.g., advice ties, friendship ties) | Network Perspective |
|  | STRENGTH - Amount of time, emotional intensity, intimacy (mutual confiding) and reciprocity of the tie | Network Perspective |
